# Krüppel-Like Factor 6 Rendered Rat Schwann Cell More Sensitive to Apoptosis via Upregulating FAS Expression

**DOI:** 10.1371/journal.pone.0082449

**Published:** 2013-12-04

**Authors:** Ting Gui, Yueming Wang, Lixing Zhang, Wenjing Wang, Hao Zhu, Wenlong Ding

**Affiliations:** 1 Department of Anatomy, Shanghai Jiao Tong University School of Medicine, Shanghai, China; 2 State Key Laboratrory of Oncogenes and Related Genes, Shanghai Cancer Institute, Renji Hospital, Shanghai Jiao Tong University School of Medicine, Shanghai, China; IISER-TVM, India

## Abstract

Krüppel-like factor 6 (KLF6) is a tumor suppressor gene and play a role in the regulation of cell proliferation and apoptosis. After the peripheral nerve injury (PNI), the microenvironment created by surrounding Schwann cells (SCs) is a critical determinant of its regenerative potential. In this study, we examined the effects of KLF6 on SCs responses during PNI. Both KLF6 mRNA and protein expression levels were upregulated in the injured sciatic nerve, and immunofluorescence results showed that many KLF6-positive cells simultaneously expressed the SC markers S-100 and p75NTR. The apoptosis inducers TNFα and cisplatin upregulated KLF6 expression in primary cultured SCs and the SC line RSC96. Although KLF6 overexpression exacerbated cisplatin- and TNFα-induced apoptosis, expression levels of the apoptosis regulators Bcl2 and Bax were not significantly affected in either KLF6-overexpressing or KLF6-depleted RSC96 cells. Realtime PCR arrays and qRT-PCR demonstrated that KLF6 overexpression upregulated four pro-apoptotic genes, FAS, TNF, TNFSF12, and PYCARD, and inhibited expression of the anti-apoptotic IL10 gene expression. Further analysis revealed that FAS protein expression was positively correlated with KLF6 expression in SCs. These data suggest that KLF6 upregulation may render SCs more vulnerable to apoptosis after injury via upregulating FAS expression.

## Introduction

The capacity of the mammalian peripheral nervous system (PNS) to regenerate after injury is robust relative to that of the central nervous system (CNS). Schwann cell (SC) surrounding the neurite is one of determinants of peripheral nerve regeneration potential [1,2]. SCs promote action potential propagation by ensheathing axons in myelin and by regulating the surface organization of axonal ionic channels [3]. Wallerian degeneration and subsequent regeneration after injury is accompanied by the proliferation and activation of SCs, which produce factors that promote axonal regrowth at the proximal nerve stump and provide a growth-permissive surface for axon guidance [4]. Transplantation of SCs at the site of injury can also promote repair in CNS demyelinating diseases and nerve regeneration after trauma, and SCs seeded in a polymer foam conduit facilitate guided peripheral nerve regeneration [5,6]. Survival of SCs in the early stage of PNI is required for functional repair, but the mechanisms determining SC survival or death remain poorly understood.

 The 17-member Krüppel-like factor (KLF) family of gene regulatory proteins all contain Cys(2)/His(2) zinc finger motifs in the carboxy terminal domain that confer preferential binding to GC/GT-rich sequences in gene promoter and enhancer regions. These zinc finger transcription factors have been implicated in many developmental processes, including proliferation, differentiation, and apoptosis [7,8]. Moore et al. screened several KLF family members for effects on neuronal regeneration and found that KLF4 knockout in retinal ganglion cells (RGCs) led to increased neurite growth *in vitro* and increased neurite regeneration after optic nerve injury *in vivo* [9]. KLF5 is immunohistochemically localized in the human prefrontal cortex and hippocampus, and has been identified as a schizophrenia-susceptibility gene [10]. Lei et al. reported a significant reduction in sensory neurons due to increased apoptosis in newborn KLF7 knockout mice [11]. Thus, different KLF family members have diverse roles in regulating the balance between apoptosis, proliferation, and regeneration in the nervous system.

During embryonic development, KLF6 is expressed in the developing forebrain and midbrain, and expression was also be detected in adult forebrain neurons [12]. Moore et al. reported that overexpression of KLF6 and KLF7 in mouse RGCs significantly increased neurite growth [9]. In retinal explants from zebrafish, both KLF6 and KLF7 were necessary for axon growth [13]. Moreover, promotion of CNS regeneration by Tuba1a was shown to depend on KLF6 and KLF7 [14]. In contrast to neurons, little is known about KLF6 expression or function in SCs. Here, we demonstrated that KLF6 expression was upregulated in SCs within the injured sciatic nerve, and that KLF6 overexpression rendered SCs more susceptible to apoptotic stress, though didn’t affect Bcl2 and Bax expression in untreated SCs. Results from PCR arrays and Western Blotting indicated that KLF6 was involved in the regulation of the death receptor mediated apoptosis pathway in SCs, and Fas is one of its downstream genes. 

## Materials and Methods

### Sciatic nerve injury model

Young adult male Sprague Dawley rats (230–270 g) were purchased from the Animal Care Facility of Shanghai Jiao Tong University School of Medicine (Shanghai, China) and housed under a 12 h:12 h light:dark cycle with *ad libitum* access to food and water. All surgical procedures were performed under aseptic conditions on animals deeply anesthetized with pentobarbital (40 mg/kg IP). An incision was made through the skin below the hip. The muscles and fascia were blunt-dissected using fine surgical scissors. The right sciatic nerve was exposed 1.0 cm distal to the sciatic notch by blunt dissection, crushed by small flat forceps at the mid-point for 10 s, and then unclamped for 10 s. Nerve crush was repeated three times to generate a transparent nerve gap while maintaining the integrity of the epineurium. After nerve injury, the skin was sutured with screen wire 4-0. As a control, the contralateral nerve was exposed but not crushed. Rats were sacrificed at 12 h, 1 d, 3 d, 7 d, 14 d, and 30 d after crush injury (n=5 animals for each time point), and the sciatic nerve spanning the crush site excised and immersed in 4% paraformaldehyde/PBS for immunofluorescence (IFC) observations. All experiments conformed to the guidelines of the Chinese Council for Animal Care and were approved by the Animal Care Committee of the Laboratory Animal at Shanghai Jiao Tong University School of Medicine.

### SCs culture

Primary SCs were obtained using our previous method [15]. Brieﬂy, sciatic nerves from 2- to 3-day-old Sprague Dawley rats were harvested and digested with 0.12% collagenase and 0.05% trypsin (Sigma-Aldrich, USA) at 37 °C for 18−25 min. Cells were re-suspended in standard growth medium (DMEM supplemented with 2 mM glutamine, 100 units/ml penicillin, 100 μg/ml streptomycin, and 10% heat-inactivated fetal bovine serum), seeded onto poly-L-lysine-coated coverslips, and allowed to adhere overnight. After 24 h, cells were treated with cytosine arabinoside (5 μg/ml; Sigma-Aldrich, USA) for 3 days to eliminate proliferating ﬁbroblasts. Following this treatment, the culture medium was replaced with fresh medium supplemented with forskolin (2 μM; Sigma-Aldrich, USA) and basic ﬁbroblast growth factor (bFGF; 20 ng/ml; Invitrogen Corporation, USA) to promote SCs proliferation. After 8 days, SCs were deplated with 0.25% EDTA-trypsin (Sigma-Aldrich, USA) and passaged. The culture medium was changed three times per week. The purity of rat primary SC cultures was determined by S100 immunoreactivity, and was > 96% for all presented studies (data not shown).

A rat Schwann cell line, RSC96 (Cell bank, Chinese Academy of Sciences) was cultured in standard growth medium at 37 °C under a humidified 5% CO_2_ atmosphere. In the cell apoptosis experiments, different doses of cisplatin (Sigma-Aldrich, USA) or TNFα (Millipore, USA) were added to the completed culture medium to reach its final concentration indicated.

### Real-time reverse transcription-polymerase chain reaction

Total RNA was extracted using TRIzol reagent (Invitrogen, USA) and reverse transcribed using the PrimeScriptTM RT Reagent Kit (Perfect Real Time) (TaKaRa Biotechnology, Japan). Realtime polymerase chain reaction (PCR) was subsequently performed as described [16]. The primers for the target genes are listed in Table S1.

### Western blotting

Proteins in cell lysates were separated by SDS-polyacrylamide gel electrophoresis and transferred onto nitrocellulose membranes. The membranes were blocked with 5% nonfat dry milk in Tris-buffered saline (TBS), washed with TBS, and incubated overnight at 4 °C with anti-KLF6 antibody (1:200, Santa Cruz, USA), anti-Bcl-2 antibody (1:200, Cell Signaling Technology, USA), anti-Bax antibody (1:200, Cell Signaling Technology, USA), anti-FAS antibody (1:200, R&D System, USA), or anti-GAPDH antibody (1:5000, Sigma-Aldrich, USA; used as a gel loading control). The membranes were then washed in TBS and incubated for 1.5 h in TBS containing 5% nonfat dry milk and a peroxidase-conjugated secondary antibody (1:3000, Sigma-Aldrich, USA). Immunolabeling was visualized using an enhanced chemiluminescence detection kit (Millipore, USA).

### Immunofluorescent staining

Sciatic nerves were fixed in 4% paraformaldehyde in PBS for 2 h at room temperature (RT), dehydrated by successive overnight incubations (4°C) in 20% sucrose in PBS and then 30% sucrose in PBS, and then embedded with OCT. Frozen sections (-25 °C) were cut at 10-μm thickness and dried overnight at RT. Sections were washed twice in PBS and blocked with a 1% BSA/0.3% Triton X100/PBS solution for 2 h at RT. Samples were incubated with anti-KLF6 (1:100, Santa Cruz, USA) and anti-S-100 (1:100, Santa Cruz, USA). After washing three times in PBS, immunolabeled sections were incubated in the corresponding FITC- or Cy3-conjugated secondary antibody (1:100, Abcam, USA) in a dark room for 2 h at 37 °C. The results of immunostaining were examined with a Leica DM2500 ﬂuorescence microscope (Germany) and an Axiovert/LSM 510 confocal scanning microscope (Carl Zeiss Microimaging, Inc., Germany).

### Caspase-3 assay

Active caspase-3 was detected using NucView™ 488 Caspase-3 Assay Kit for Live Cells (Biotium, USA). Primary SCs were washed twice with PBS, fixed with 4% paraformaldehyde in PBS for 1 h at RT, and incubated in fresh culture medium containing 5 μM NucView 488 substrate stock solution at RT for 30 min. Cultures were then counterstained with Hoechst 33342, washed with PBS, and examined by fluorescence microscopy.

### TUNEL assay

Terminal deoxynucleotidyl transferase UTP-biotin nick end labeling (TUNEL) was performed using the In Situ Cell Death Detection Kit (Roche, Switzerland) according to the manufacturer’s instructions. Cells of the RSC96 line were harvested, washed twice with PBS, and fixed with 4% paraformaldehyde in PBS for 1 h at RT. Cells were then washed twice in PBS and permeabilized with 0.1% Triton X-100 for 2 min on ice followed by incubation with TUNEL assay reagent and Hoechst 33342 for 1 h at 37 °C. Cells were washed twice with PBS and TUNEL-positive cells quantified by fluorescence microscopy. All experiments were performed in triplicate.

### Cell transfection

Packaged lentiviral virus KLF6-pGC-FU-GFP-LV and corresponding control virus were purchased from GENECHEM (Shanghai, China) and viral titers determined. RSC96 cells were seeded at a density of 2000/well in 96-well plates. After 24 h, cells were inoculated with virus particles at a multiplicity of infection (MOI) of 50 in the presence of 6 μg/ml polybrene. Transfection efﬁciency was routinely ≈90% as indicated by the fraction of GFP-positive cells.

### RNA Interference-based gene knockdown

A small interfering RNA (siRNA) targeting KLF6 and the negative control siRNA (NC) were purchased from Sigma-Aldrich (USA). RSC96 cells were incubated in serum-free media for 8−12 h before transfection. Cells were transfected with siRNA oligos using an NEPA21 electroporator (Nepa gene, JP) according to the manufacturer’s instructions. Cells were harvested 24 h after transfection for real-time PCR and Western blotting to determine the efficiency of gene knockdown.

### RT^2^ profiler PCR arrays

Total RNA from RSC96 cells stably transfected with KLF6-pGC-FU-GFP-LV or control vector was extracted and analyzed by the Apoptosis PCR Array (SA Biosciences PAHS-012Z) according to the manufacturer’s instructions (SA Biosciences, Frederick, MD) on an Applied Biosystems 7500 Fast Real-Time PCR System. Expression of each gene was normalized to the mean Ct value of four housekeeping genes in the PCR array (β2-microglobulin, β-actin ribosomal protein L13A, hypoxanthine phosphorybosyl transferase-1, and β-actin) and then to the pre-treatment expression level to determine the fold-change. Relative fold-change in expression was calculated by the ΔΔCt method and the values are expressed as 2^-ΔΔCt^. All data points are the means of duplicate trials.

### Statistical analysis

Data are presented as the mean ± SD. Means were compared by Student’s *t*-tests. A *p* < 0.05 was considered statistically significant.

## Results

### KLF6 expression is upregulated in the injured sciatic nerve

**Figure 1:**
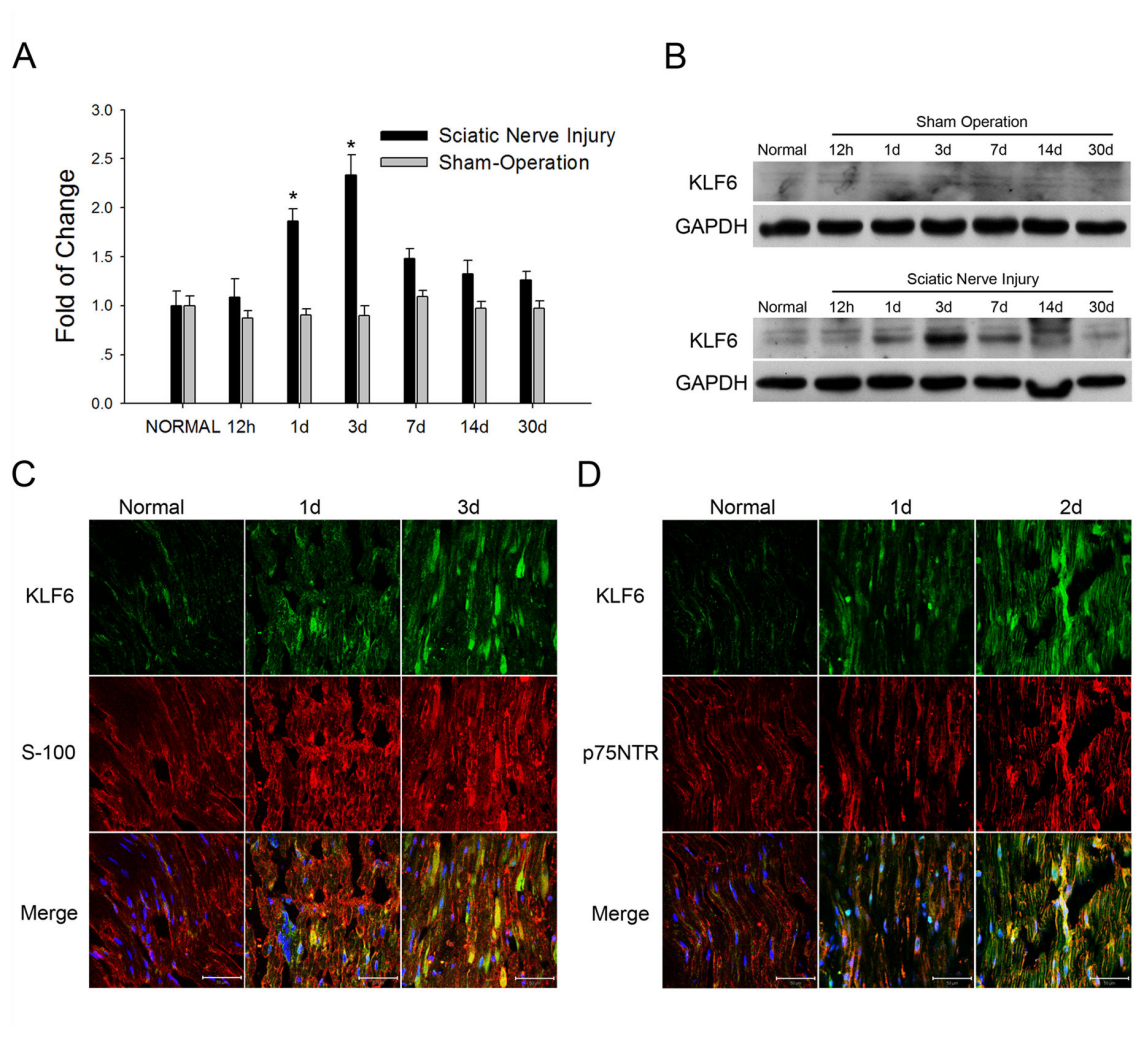
KLF6 expression was induced in injured sciatic nerves. *A*. Time course of KLF6 expression as indicated by real-time PCR following rat sciatic nerve injury. The data are normalized to GAPDH expression and presented as fold-change relative to the normal control. *p<0.05 vs. normal control (*t*-test). *B*. Western blots showed that KLF6 expression was upregulated in a time dependent manner at the site of sciatic nerve injury, while in the sham operation group KLF6 expression showed no obvious change. *C*. and *D*. Immunohistochemical detection of KLF6, S-100 and p75NTR expression in the injured nerve. Representative images are shown and the scale bar=50 μm.

To explore the effects of different KLF family members on regeneration following PNI, we established a rat sciatic nerve injury (SNI) model and screened injured nerves for expression of KLFs genes. We found that expression of KLF6 mRNA was upregulated in the injured nerve compared to the contralateral sham-operation nerve (Figure S1A). We then measured changes in KLF6 mRNA at multiple time points up to 30 days after SNI by realtime PCR. KLF6 expression was upregulated in injured nerves on day 1 post-injury and reached peak expression on day 3 (Figure 1A), then began to decrease on 7 day and reached near normal levels by day 30, indicating that KLF6 may regulate the early neuronal or SC response to SNI. Western blots revealed that KLF6 protein expression was also transiently upregulated following SNI (Figure 1B). 

We then determined the cellular and subcellular location of KLF6 expression after SNI by immunofluorescence staining. Elevated KLF6 expression was detected as early as 12 h after injury in both the distal and proximal portions of the injured nerve (Figure S1B). The number of KLF6-positive cells increased with time after injury, in accordance with Western blotting results. And in the injury nerves, KLF6 protein could be detected in the cell nuclear as well as cytoplasm. Among these KLF6-positive cells, many were also immunopositive for S-100 and p75NTR proteins, which are SC-specific markers in the peripheral nerve system (Figure 1C and 1D), indicating that KLF6 expression was upregulated in the SCs in the injured sciatic nerve.

### KLF6 expression was enhanced after apoptosis agent treatment in SCs

The survival of SCs is a critical determinant of peripheral nerve regeneration. Chen et al. reported that after SNI, SC number decreased due to apoptosis [17]. We found that, in the injured sciatic nerve, nuclear fragmentation could be observed in some cells in both the distal and proximal portions (data not shown). And TUNEL analysis demonstrated that apoptotic cells could be detected 1 day after injury and cell number peaked around day 3 (Figure S2). We therefore examined the relationship between KLF6 expression and SC apoptosis. 

**Figure 2:**
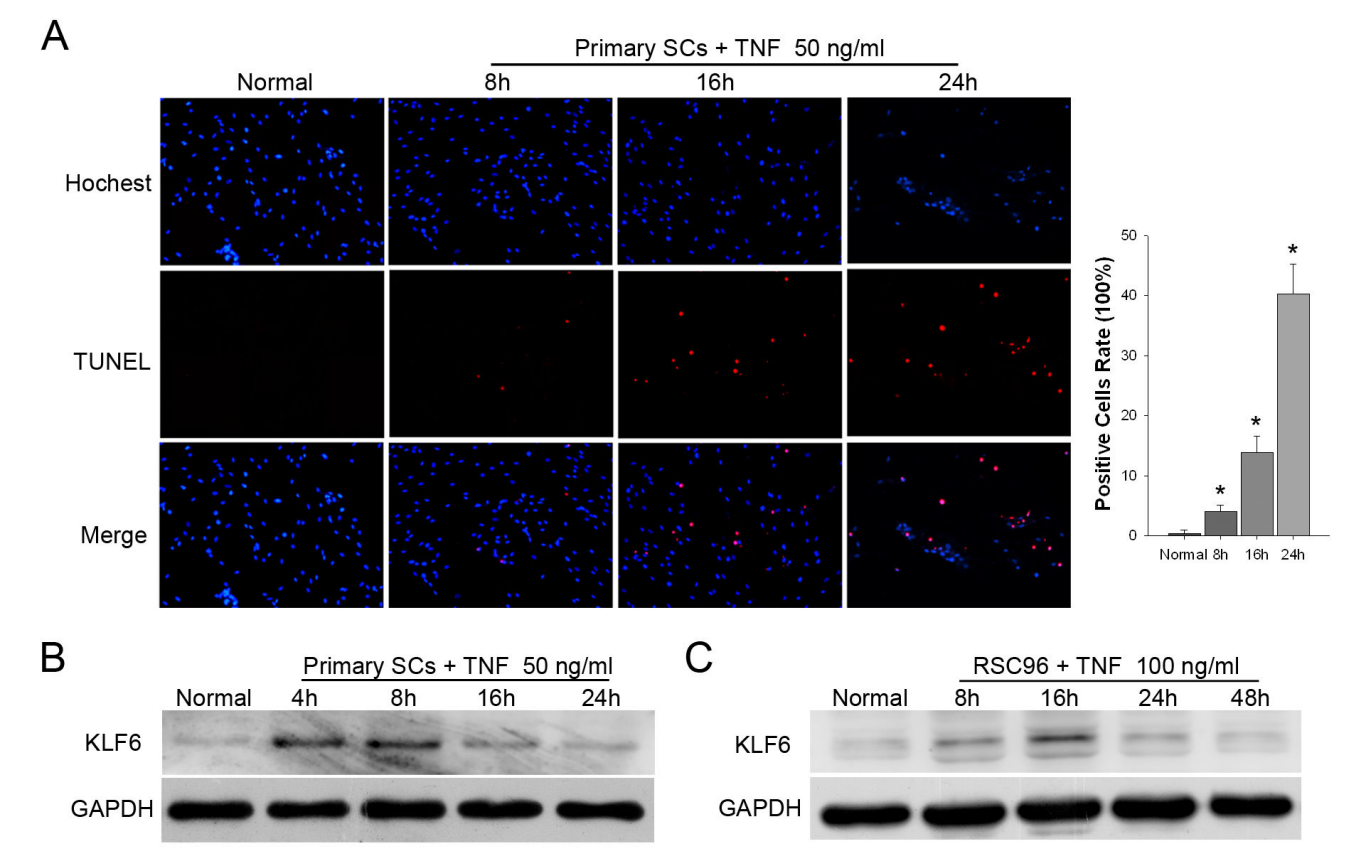
TNFα treatment promoted KLF6 expression in SCs. *A*. primary SCs were treated with 50ng/ml TNFα for time indicated and subjected to TUNEL staining. Representative images are shown along with the quantification of 5 randomly selected fields. Original magnification, 200×; *p<0.01 vs. normal (*t*-test). *B*. Western Blotting of KLF6 expression in primary SCs or RSC96 cells (*C*) treated with TNFα.

Tumor necrosis factor-α (TNFα) is a multipotent inflammatory cytokine that induces a wide variety of responses including apoptosis and proliferation, Yuan et al. reported that low concentration of TNFα induced SCs proliferation, while high concentration of TNFα induced SCs apoptosis *in vitro* [18]. Here, TUNEL assay was used to detect apoptotic cells in the primary SCs treated with high concentration of TNFα (50 ng/ml). The results showed that apoptotic cells emerged 8 hour after TNFα treatment, and the apoptotic SCs number increased in a time-dependent manner (Figure 2A). Simultaneously, KLF6 protein expression was upregulated after TNFα treated for 4 hours, and reached peak at 8 hour (Figure 2B). A similar transient change in KLF6 expression was also observed in RSC96 cells (Figure 2C).

**Figure 3:**
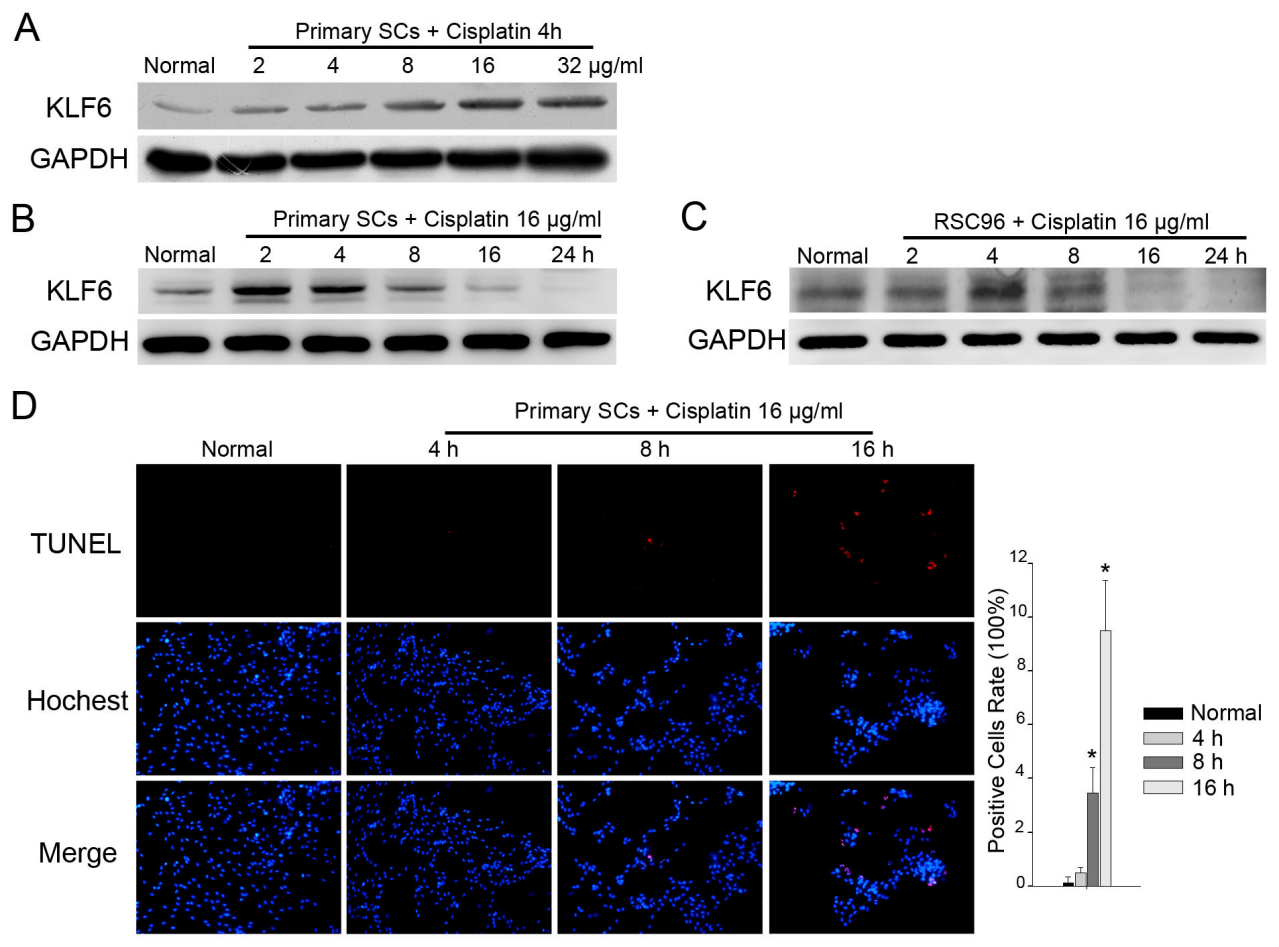
Cisplatin treatment promoted KLF6 expression in SCs. *A*. Western blotting of KLF6 expression in primary SCs treated with different doses of cisplatin. *B* and *C*. Primary SCs and the SC cell line RSC96 were treated with 16 μg/ml cisplatin and KLF6 protein expression measured at several time points. *D*. A 16 hours time course experiment on SCs treated with 16 μg/ml cisplatin, and then SCs were subjected to TUNEL staining. Representative images are shown along with the quantification of 5 randomly selected fields. Original magnification, 200×; *p<0.01 vs. untreated cells at 0 h (*t*-test).

To further investigate the relationship of KLF6 expression and SCs apoptosis, primary SCs were then exposed to cisplatin, a DNA-damaging agent that induced apoptosis in many cell types. Different doses of cisplatin were added to the culture medium for 4 hours, and KLF6 expression was detected by Western blot. We found that 8 μg/ml cisplatin caused a measurable increase in KLF6, with maximum induction at 32 μg/ml (Figure 3A). We then investigated temporal changes in KLF6 expression in response to a medium dose of 16 μg/ml cisplatin (Figure 3B). Expression of KLF6 increased within 2 h of cisplatin exposure, followed by a striking downregulation after 8 h. A similar transient change in KLF6 expression was also observed in RSC96 cells (Figure 3C), and these changes were correlated with the activation of the apoptosis effector caspase-3 (Figure S3) and the cell apoptosis (Figure 3D; *p*<0.01 *vs.* baseline). The fraction of primary SCs positive for TUNEL staining increased from 0.13±0.22% at baseline to 9.49±1.8% after 16 h of cisplatin treatment. These results demonstrated that the TNFα and cisplatin induced SCs apoptosis and upregulated KLF6 expression.

### KLF6 overexpression renders SCs more vulnerable to apoptosis

**Figure 4:**
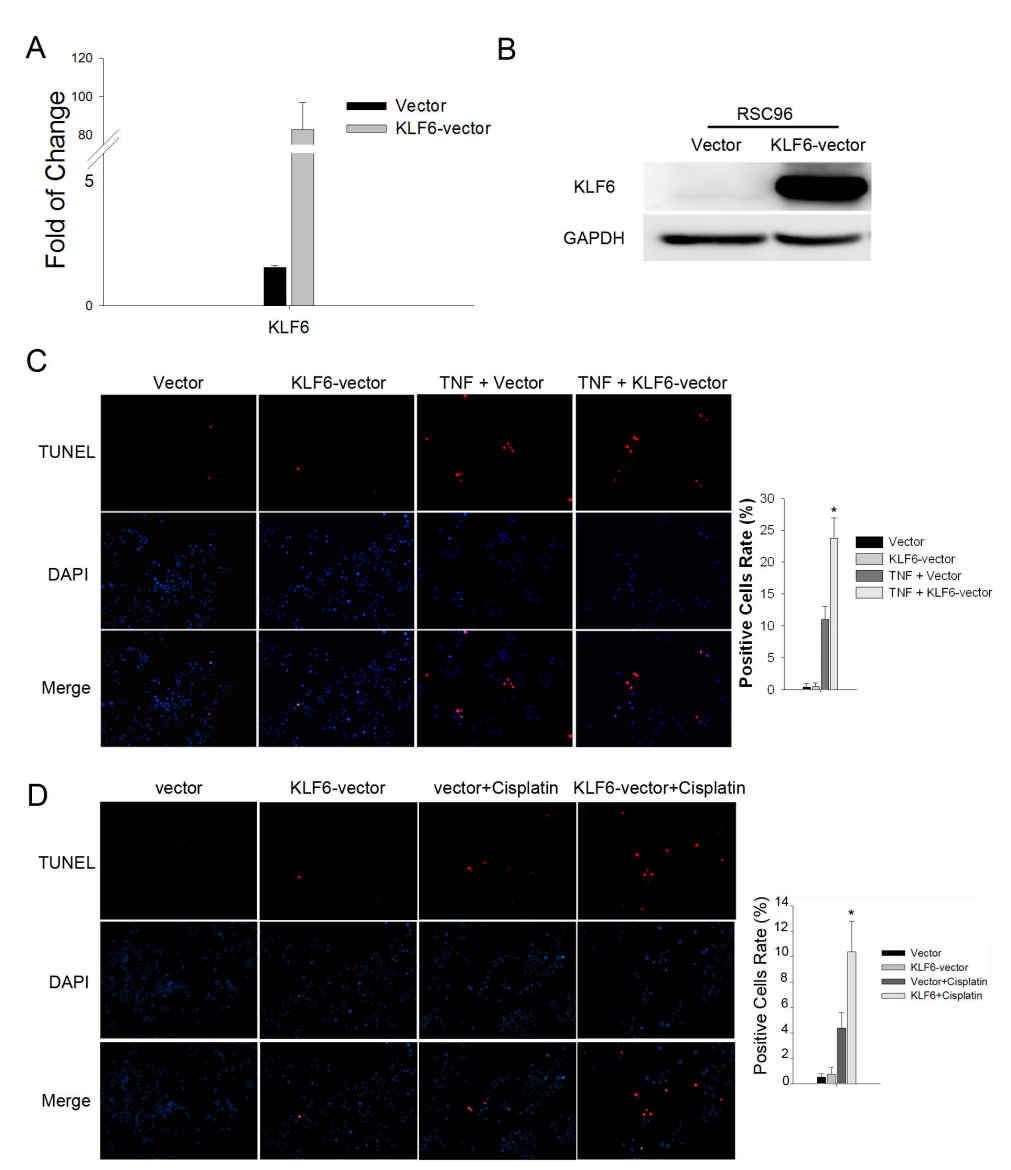
KLF6 rendered SCs more susceptible to apoptotic stress. *A*. KLF6 mRNA expression analyzed by real-time PCR in RSC96 cells overexpressing KLF6 (KLF6-vector) and RSC96 cells stably transfected with the empty vector (Vector). *p<0.05 vs. Vector (*t*-test). *B*. Western blot of KLF6 expression in RSC96 cells. *C*. RSC96 cells stably transfected with control vector control or overexpressing KLF6 were treated with 100 ng/ml TNFα for 48 hours and then subjected to TUNEL staining. Representative images are shown along with the quantification of 5 randomly selected fields. Original magnification, 200×; *p<0.05 vs. the TNF+vector group (*t*-test). *D*. RSC96 cells were treated with 16 μg/ml cisplatin for 8 hours and then subjected to TUNEL staining. Representative images are shown along with the quantification of 5 randomly selected fields. Original magnification, 200×; *p<0.05 vs. the Vector + Cisplatin group (t-test).

To illuminate the function of KLF6 in SC apoptosis, we established a stable RSC96 cell line overexpressing KLF6 through infection with a lentivirus vector (pGC-FU-GFP-LV) (Figure 4A and 4B). RSC96 cells overexpressing KLF6 exhibited enhanced apoptosis in response to 48 h TNFα treatment compared to RSC96 cells stably expressing the empty vector (RSC96-vector) as revealed by TUNEL staining (23.76 ± 3.20% vs. 11.04 ± 2.00%, *p*<0.05), while there was no difference in the rate of spontaneous apoptosis between RSC96-KLF6 and RSC96-vector cultures (0.43 ± 0.58% vs. 0.48 ± 0.59%, *p*>0.05) (Figure 4C). A similar result could also be obtained in the KLF6-overexpresing RSC96 cells treated with Cisplatin (Figure 4D), indicating that KLF6 upregulation may rendered SCs more vulnerable to apoptosis after injury.

### KLF6 regulates the death receptor-mediated apoptosis pathway

**Figure 5:**
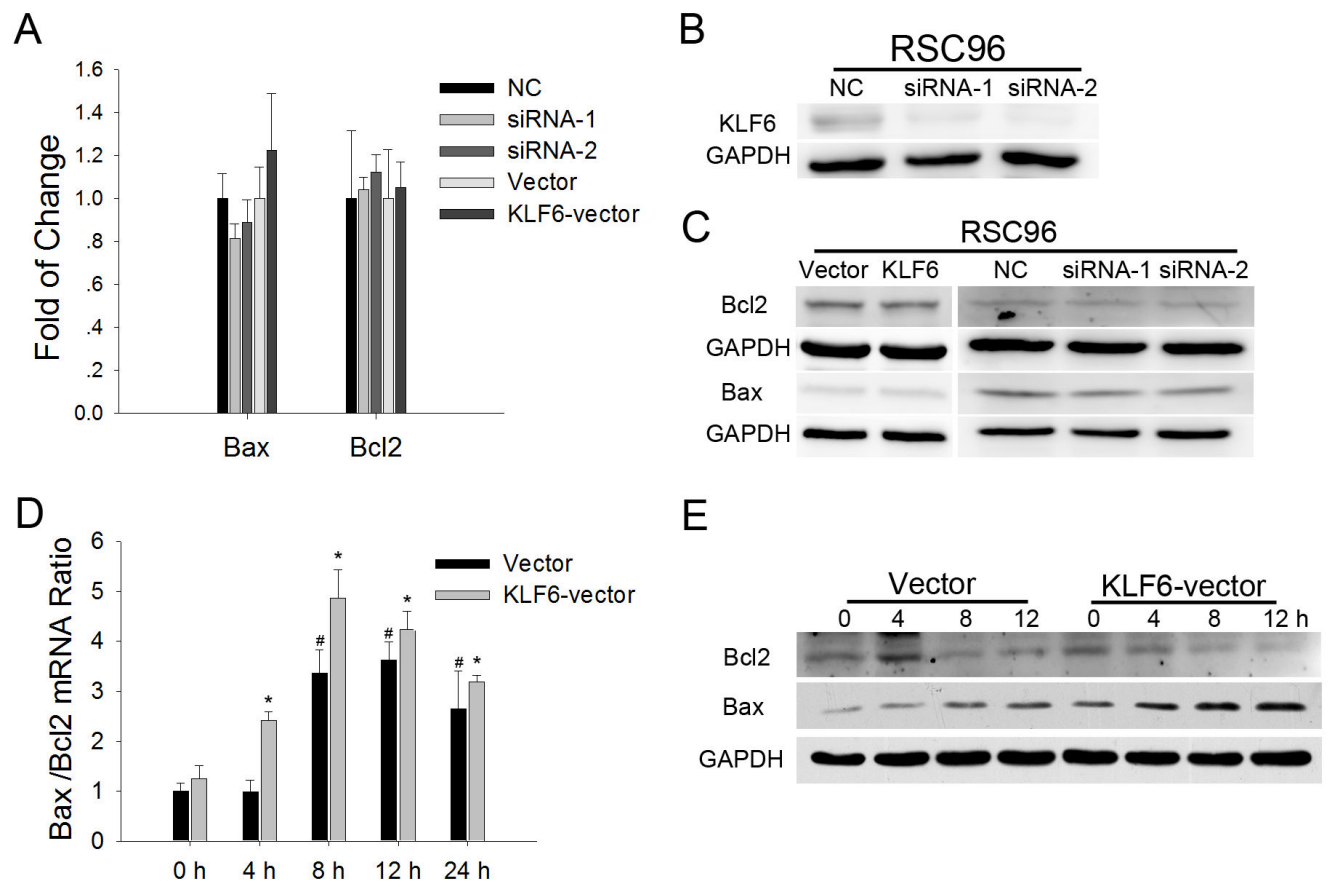
Bcl2 and Bax expression in RSC96 cells with KLF6 kncokdown or forced expression. *A*. Bcl2 and Bax expression analyzed by real-time PCR in RSC96 cells with KLF6 knockdown or overexpression. *B* and *C*. Western blot of KLF6, Bcl2 and Bax expression in RSC96 cells with KLF6 knockdown or overexpression. *D*. Bax and Bcl2 expression levels were analyzed by real-time PCR. The Bax/Bcl2 ratio increased after cisplatin treatment. *p<0.05 *vs*. untreated cells at 0 h (*t*-test). *E*. Bcl2 and Bax expression levels over 12 h as analyzed by Western blotting in RSC96 cells overexpressing KLF6.

Bax and Bcl2 proteins are ubiquitous regulators of apoptosis. However, neither Bcl2 nor Bax mRNA and protein expression levels were affected in KLF6 overexpressing RSC96 cells (Figure 5A and 5C) and basal Bax/Bcl2 mRNA ratio was not significantly different between RSC96-KLF6 cells and RSC96-vector controls (Figure 5D). However, cisplatin treatment for 4 hours did cause a significant increase in the Bax/Bcl2 mRNA ratio in RSC96-KLF6 SCs. Western blots indicated that Bax protein expression began to increase at hour 4 in RSC96-KLF6 cells, whereas increased Bax expression was not observed until hour 8 in RSC96-vector SCs. Moreover, the magnitude of Bax expression was higher in RSC96-KLF6 cells (Figure 5E). In contrast to (pro-apoptotic protein) Bax, expression of the anti-apoptotic protein Bcl2 was downregulated after cisplatin treatment for 8 hours in both RSC96-KFL6 and RSC96-vector SC lines, and its downregulation was more obvious in the RSC96-KLF6 cells at hour 12. We also examined Bax and Bcl2 expression in RSC96 cells with KLF6 depletion. Small interfering RNAs specifically targeting KLF6 were synthesized, and the knockdown efficiency confirmed (Figure 5B). As shown in Figure 5A and 5C, KLF6 knockdown did not significantly affect Bcl2 and Bax genes expression. 

**Figure 6:**
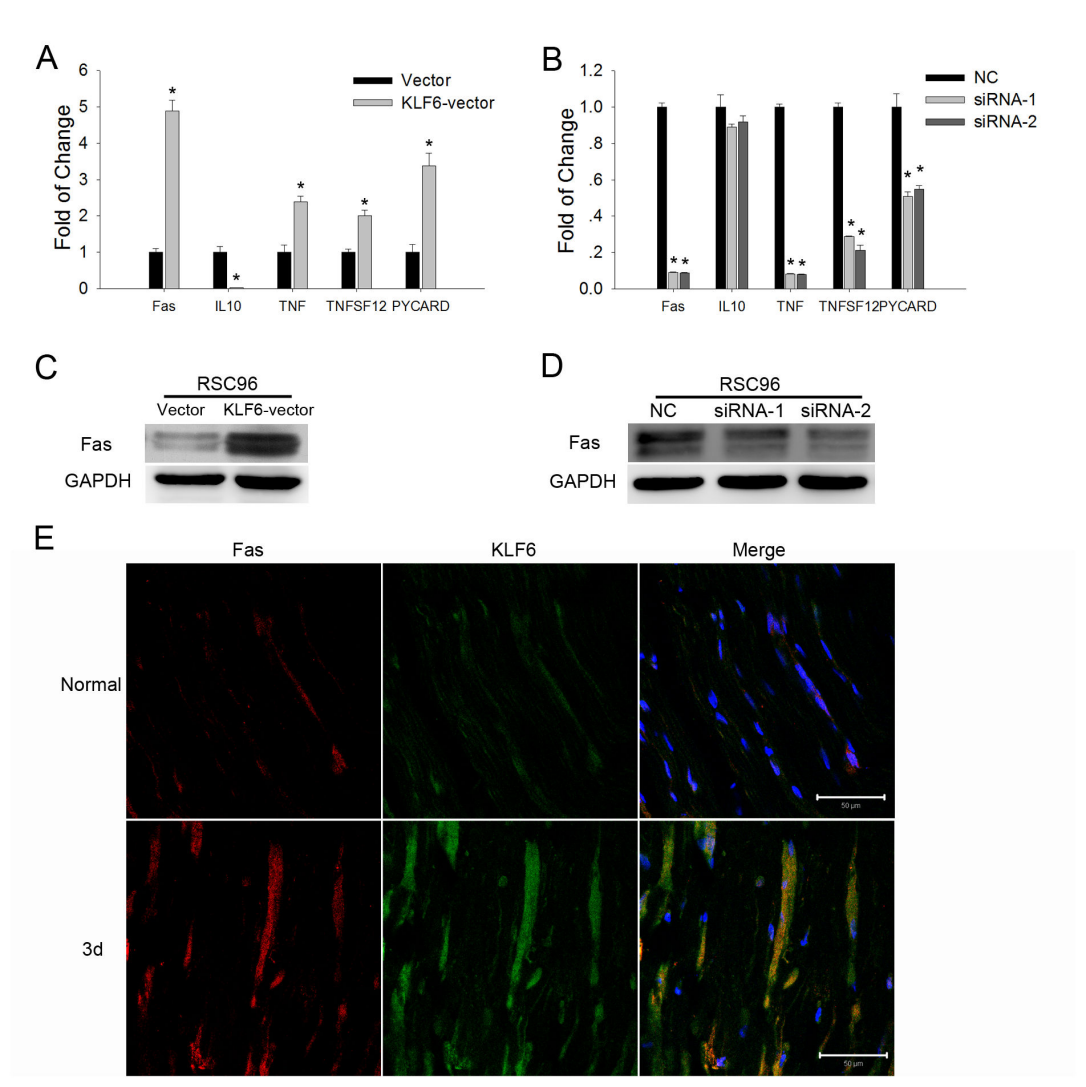
KLF6 differentially regulated apoptosis-related gene expression in SCs. *A*. Real-time PCR of apoptosis-related gene expression in RSC96 cells overexpressing KLF6. *p<0.05 *vs*. Vector (*t*-test). *B*. Real-time PCR assay of apoptosis-related gene expression in RSC96 cells with KLF6 depletion (knockout). *p<0.05 *vs*. Negative control (NC) (*t*-test). *C* and *D*. Western blots showing FAS expression in RSC96 cells overexpressing KLF6 or with KLF6 depletion. *E*. Immunohistochemical detection of KLF6 and FAS expression in the injured nerve. Representative images are shown and the scale bar=50 μm.

To further explore the apoptotic signaling pathway regulated by KLF6, we employed the Rat RT^2^ Profiler PCR Array for Apoptosis. Overexpression of KLF6 in RSC96 cells upregulated several genes involved in apoptosis: FAS (6.71-fold *vs.* the control), TNF (4.78-fold), TNFSF12 (2.24-fold), and PYCARD (2.47-fold), but downregulated expression of the anti-apoptotic factor IL10 (4.73-fold decrease) (Table 1). These results were confirmed by realtime PCR (Figure 6A). After KLF6 knockdown in RSC96 cells, FAS, TNF, TNFSF12, and PYCARD gene expression levels were downregulated, while IL10 gene expression showed no significant change (Figure 6B). We then analyzed changes in FAS protein expression by Western blotting. Overexpression of KLF6 promoted FAS protein expression, while KLF6 knockdown led to downregulation of FAS protein expression (Figure 6C and 6D). And immunofluorescence results showed that KLF6-positive cells in the injured sciatic nerve also expressed Fas protein (Figure 6E). These results suggested that KLF6 may promote apoptosis through a death receptor related signaling pathway.

**Table 1:** Realtime PCR array analysis of differentially expressed genes in KLF6 overexpressed RSC96 cells.

Name of Gene	Description	Fold up- or down-regulation (exp/control)	*p*-Value
FAS	TNF receptor superfamily, member 6	6.17 ± 1.04	<0.05
TNF	tumor necrosis factor	5.26 ± 0.63	<0.01
TNFSF12	TNF(ligand) superfamily, member 12	2.21 ± 0.20	<0.01
PYCARD	PYD and CARD domain containing	2.93 ± 0.67	<0.05
IL10	interleukin 10	-4.44 ± 0.64	<0.05

The expression of apoptosis-related genes in KLF6 overexpressed RSC96 cells were examined with PCR array, the experiments were repeated for 3 times and statistical analyses were performed. All genes with defined the threshold of 2-fold differential expression and *p*<0.05 were listed.

## Discussion

Krüppel-like factor 6 is a widely expressed zinc finger transcription factor induced as an immediate-early gene after injury. Ratziu et al. reported that KLF6 protein expression was upregulated in stellate cells 3 hours after liver injury [19], and subsequently induced transcription of TGFβ and its receptors [20]. Botella et al. found that KLF6 expression was rapidly induced in the endothelium following carotid balloon injury in rats and transactivated TGFβ-stimulated genes during vascular repair [21]. The binding of KLF6 to the iNOS promoter was rapidly increased in primary T lymphocytes subjected to NaCN-induced hypoxia, heat shock, or serum starvation, leading to increased expression of the endogenous iNOS gene and greater NO release [22]. We found that KLF6 mRNA and protein expression began to increase in the SNI one day after injury and reached a peak by day 3, indicating KLF6 functions in the early stage of SNI. Immunofluorescence results showed that many KLF6-positive cells also expressed the SC-specific marker protein S-100 and p75NTR, suggesting that KLF6 may regulate the early SC response to injury. In accordance with Chen et al. report [17], TUNEL analysis results showed that apoptotic cells in the sciatic nerve also increased 1 day after injury, and reached a peak at around 3 day, indicating a relationship between KLF6 upregulation and cell apoptosis. Thus, a preponderance of evidence implicated a role of KLF6 in the early response to injury. Considering the pro-growth effects of KLF6 reported in the nerve of CNS, KLF6 may have both beneficial and detrimental effects in the PNS, possibly promoting neurite extension but increasing the propensity for SC apoptosis after nerve injury.

However, many reports on the effects of KLF6 in cell apoptosis have been contradictory, with both pro- and anti-apoptotic responses observed depending on the tissue under study [23]. In the liver, Ghiassi-Nejad et al. reported that stellate cells overexpressing KLF6 were more susceptible to apoptotic stress as indicated by PARP cleavage [24]. Overexpression of KLF6 also induced apoptosis in several human cancers, including prostate cancer, non-small cell lung cancer, and osteosarcoma. The KLF6 protein itself was degraded when cancer cells underwent apoptosis [25–28]. Conversely, D’Astolfo et al. found that siRNA-mediated silencing of KLF6 in hepatocarcinoma and HeLa cells led to cell cycle arrest and sensitized cells to apoptosis induced by DNA damage [29]. Similarly, Salma and McDermott reported that silencing KLF6 expression enhanced apoptosis of primary hippocampal neurons [30]. We demonstrated that KLF6 mRNA and protein levels were rapidly and transiently increased in SCs within several hours during cisplatin or TNFα treatment. Cisplatin and TNFα treatment also induced SC apoptosis, suggesting that KLF6 may be involved in the regulation of SC apoptosis. A SC cell line RSC96 stably overexpressing KLF6 showed enhanced sensitivity to cisplatin or TNFα induced apoptosis as indicated by higher numbers of TUNEL-positive cells, though the apoptosis cells number between the KLF6-overexpressing group and the control showed no significantly difference. Thus, KLF6 upregulation renders SCs more vulnerable to apoptosis induced by pro-apoptosis agents, while KLF6 overexpression may not directly induce apoptosis. However, considering that cell lines may differ from the *in vivo* situation in some aspects, a further investigation of KLF6 pro-apoptosis effects in SCs *in vivo* is still necessary. 

The mitochondrial apoptotic pathway is regulated by the Bcl2 superfamily of proteins [31,32]. The Bax/Bcl2 ratio reflects the propensity or resistance to apoptosis [33,34]. However, KLF6 overexpression or depletion in SCs did not significantly affect Bax or Bcl2 mRNA and protein expression in the absence of cisplatin or TNFα, suggesting that KLF6 regulates apoptosis through an alternative pathway. Indeed, PCR array analysis revealed that KLF6 overexpression triggered the upregulation of TNF superfamily members FAS, TNF, and TNFSF12 gene expression. The PYCARD gene has been found to suppress the proliferation of thyroid cancer cells by inducing apoptosis [35], which expression was also upregulated in RSC96-KLF6 cells. Conversely, KLF6 knockdown led to downregulation of these genes, indicating that KLF6 may regulate apoptosis through a death receptor pathway. In contrast to these effects on TNF family members and PYCARD, KLF6 upregulation led to the suppression of IL10, a repressor of FAS-induced apoptosis in mouse intestinal epithelial cells [36]. 

The FAS (APO-1/ CD95) protein is a member of the death receptor (DR) family. Wohlleben et al. reported that FAS and FASL were expressed on rat SCs and may contribute to the elimination of invading autoreactive T cells in a rat model of experimental autoimmune neuritis [37]. Chen et al. found that FAS-positive SCs in the transected rat sciatic nerve were more numerous than in uninjured nerves [17]. Mimouni-Rongy et al. reported that FasL can act as a signal-transducing molecule in SCs and may contribute to peripheral nerve regeneration [38]. We demonstrated a positive correlation between FAS and KLF6 expression in RSC96 cells, and Fas protein positive cells in the injured sciatic nerve also showed KLF6 expression upregulated, strongly suggesting that FAS is a KLF6 downstream gene and mediates the pro-apoptotic effects observed.

In addition to apoptosis, FAS and TNFα have both participated in regulation of inflammation. In the PNS, TNFα is a primary mediator of inflammatory responses and in mainly synthesized and, released by SCs [39]. FASL/FAS signaling promotes inflammatory demyelination Following peripheral nerve damage [40,41]. IL10 inhibits antigen presentation, cytokine expression, and T helper cell function during the inflammatory response [42]. In SCs, KLF6 upregulated FAS and TNF gene expression and downregulated IL10 gene expression, suggesting that KLF6 may also participate in the inflammation response in SCs. Further studies are necessary to delineate the relationship between KLF6 expression and inflammation of damaged peripheral nerves.

In conclusion, KLF6 expression was upregulated in SCs after SNI. Overexpression of KLF6 rendered SCs more vulnerable to pro-apoptotic agents *in vitro*. The FAS gene appears to be a KLF6 downstream gene and so may mediate the pro-apoptotic effect of KLF6 overexpression.

## Supporting Information

Figure S1 **KLF6 mRNA expression was upregulated in injured sciatic nerves.** *A*. Realtime PCR analysis of several members of KLF superfamily transcription factor genes expression in injured sciatic nerves compared with which in the sham-operation group. The KLFs genes tested here including KLF1, KLF3, KLF4, KLF5, KLF6, KLF7, KLF9, KLF10, KLF11, KLF12, KLF15 and KLF16. *B*. Immunohistochemical detection of KLF6 and S-100 expression in the injured nerve. Representative images are shown.
(TIF)

Figure S2 **Apoptosis cell number increased in injured sciatic nerve tissue.** Rat sciatic nerve samples after injury at several time points indicated were subjected to TUNEL assay. Representative images are shown along with the quantification of 5 randomly selected fields.. Original magnification, 200×.
(TIF)

Figure S3 **Actived Caspase-3 positive SCs increased in primary Schwann cells treated with cisplatin.** *A*. 16 hours time course experiment on SCs treated with 16 μg/ml cisplatin, NucView 488 substrate (green channel) was added at the indicated time points. Representative images are shown along with the quantification of 5 randomly selected fields. Original magnification, 200×; *p<0.01 vs. untreated cells at 0 h (*t*-test).
(TIF)

Table S1 **The sequences of gene-specific primers used for qRT-PCR.**
(DOC)
