# Supplementary figures and images for: Krüppel-Like Factor 6 Rendered Rat Schwann Cell More Sensitive to Apoptosis via Upregulating FAS Expression
Source: PLoS One. 2013 Dec 4;8(12):e82449. doi: 10.1371/journal.pone.0082449 (PMC3853331; doi:10.1371/journal.pone.0082449)

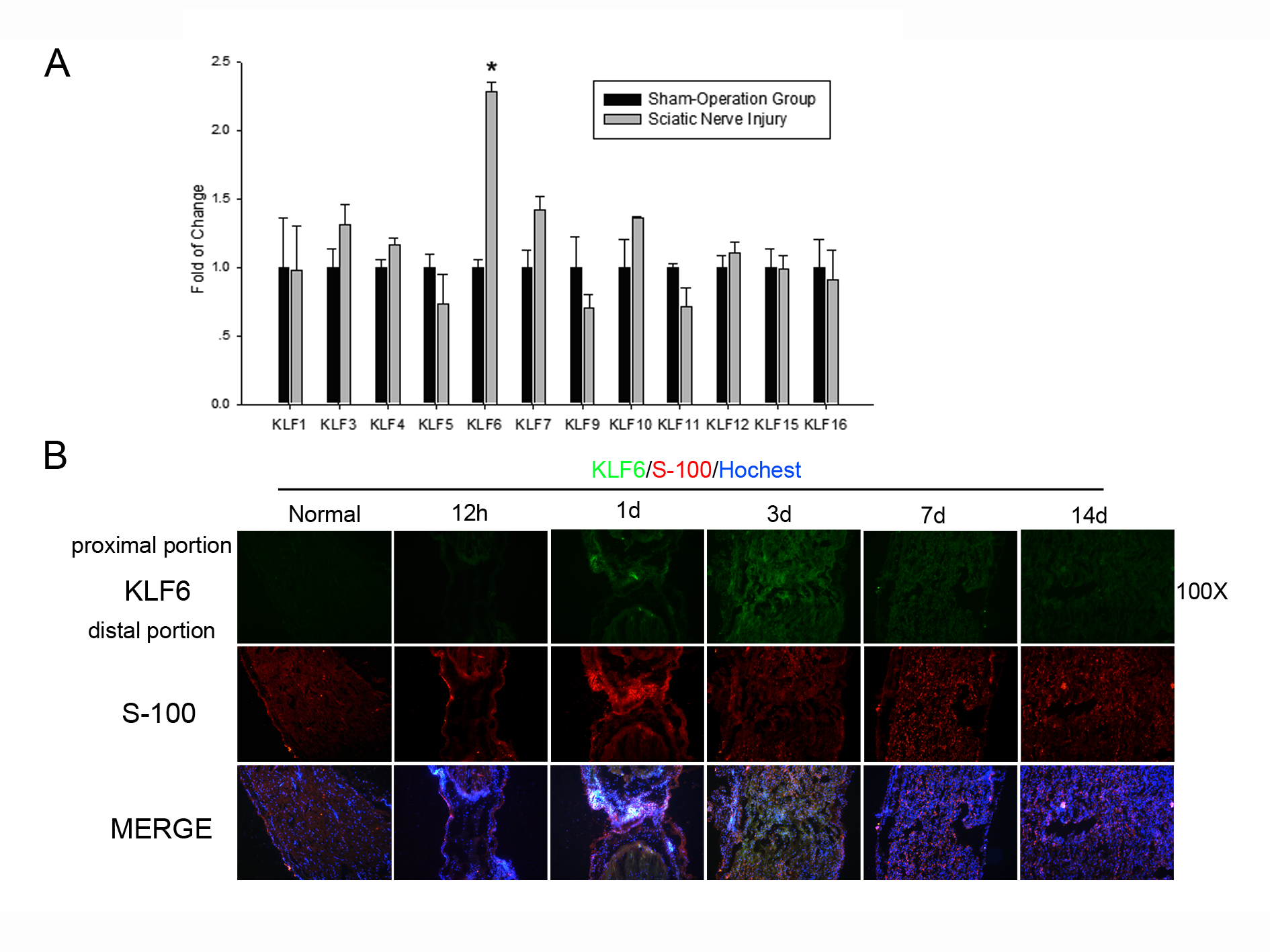

Supplement: Figure S1 — KLF6 mRNA expression was upregulated in injured sciatic nerves. A. Realtime PCR analysis of several members of KLF superfamily transcription factor genes expression in injured sciatic nerves compared with which in the sham-operation group. The KLFs genes tested here including KLF1, KLF3, KLF4, KLF5, KLF6, KLF7, KLF9, KLF10, KLF11, KLF12, KLF15 and KLF16. B. Immunohistochemical detection of KLF6 and S-100 expression in the injured nerve. Representative images are shown. (TIF) [file pone.0082449.s001.tif]

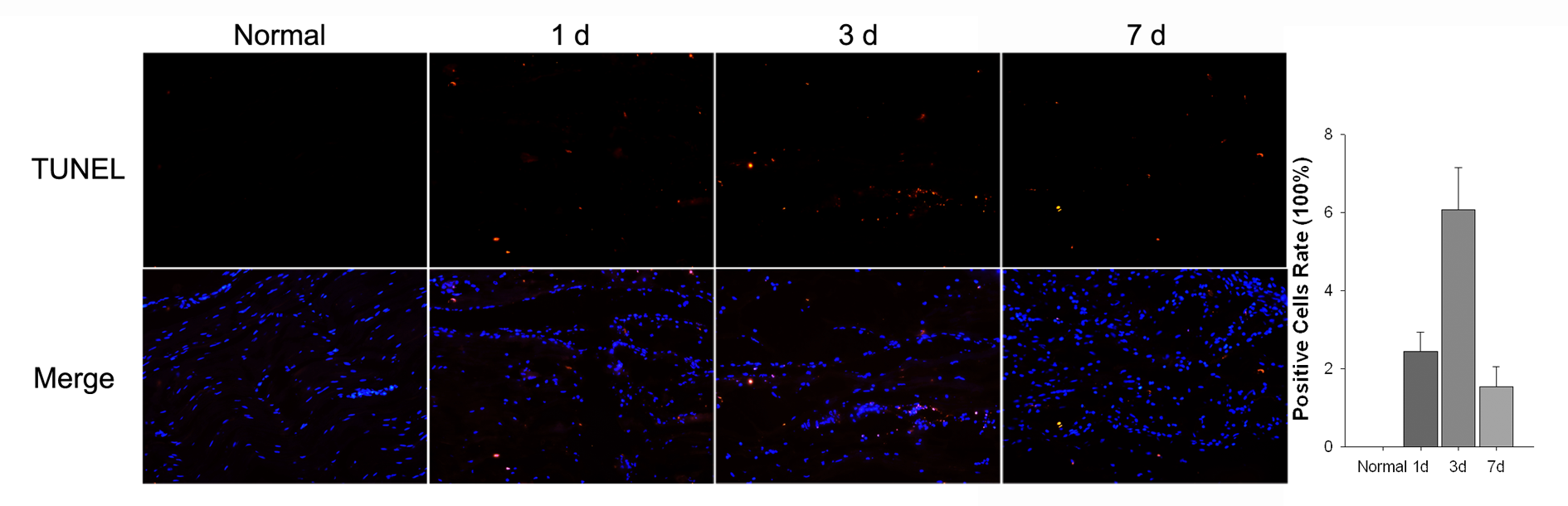

Supplement: Figure S2 — Apoptosis cell number increased in injured sciatic nerve tissue. Rat sciatic nerve samples after injury at several time points indicated were subjected to TUNEL assay. Representative images are shown along with the quantification of 5 randomly selected fields.. Original magnification, 200×. (TIF) [file pone.0082449.s002.tif]

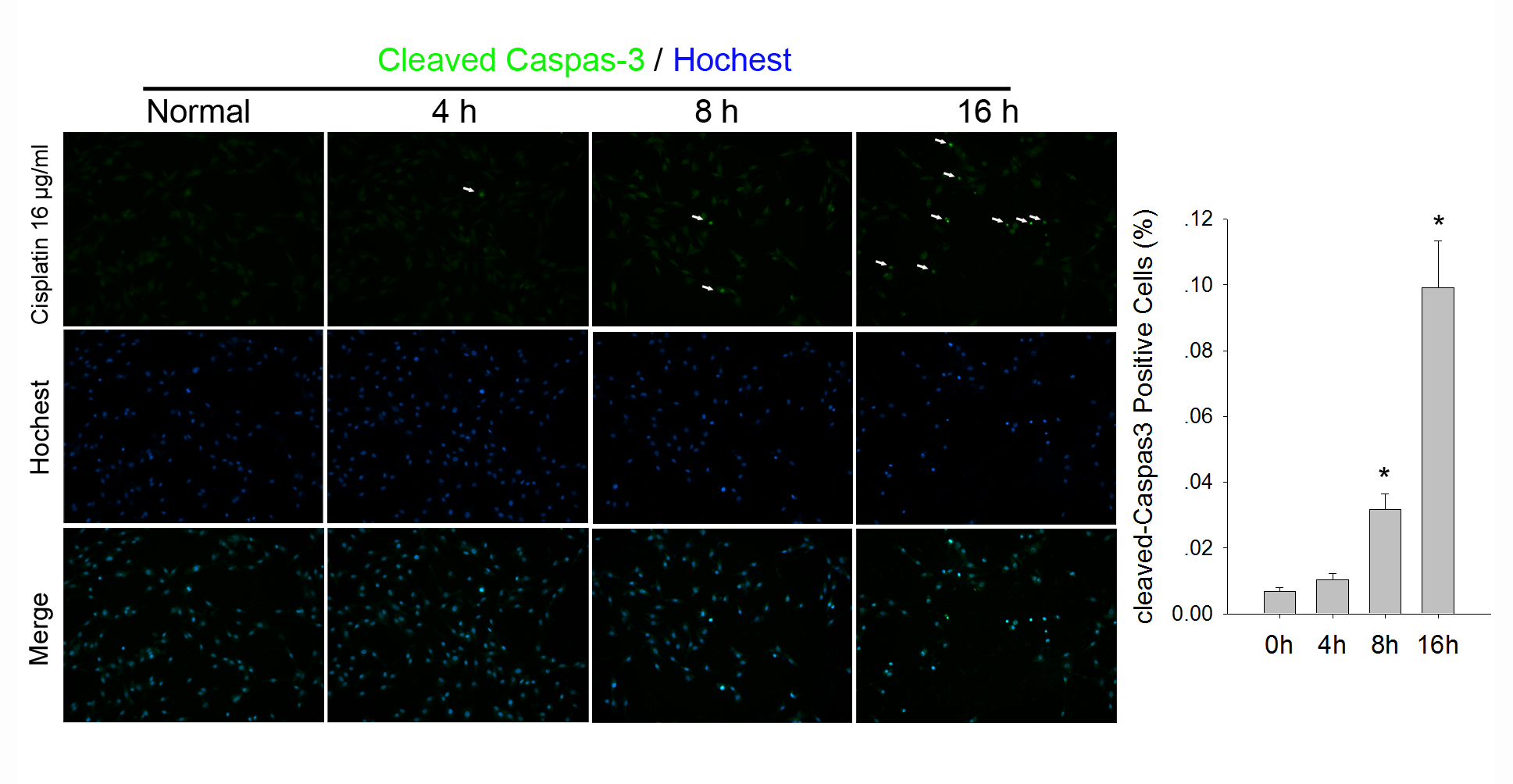

Supplement: Figure S3 — Actived Caspase-3 positive SCs increased in primary Schwann cells treated with cisplatin. A. 16 hours time course experiment on SCs treated with 16 μg/ml cisplatin, NucView 488 substrate (green channel) was added at the indicated time points. Representative images are shown along with the quantification of 5 randomly selected fields. Original magnification, 200×; *p<0.01 vs. untreated cells at 0 h (t-test). (TIF) [file pone.0082449.s003.tif]
