# Supplementary material for: Krüppel-Like Factor 6 Rendered Rat Schwann Cell More Sensitive to Apoptosis via Upregulating FAS Expression
Source: PLoS One. 2013 Dec 4;8(12):e82449. doi: 10.1371/journal.pone.0082449 (PMC3853331; doi:10.1371/journal.pone.0082449)
Supplement: Table S1 — The sequences of gene-specific primers used for qRT-PCR. (DOC) [file pone.0082449.s004.doc]

Table S1. The sequences of gene-specific primers used for qRT-PCR

| **Gene name** | **Forward (5'-3')** | **Reverse (5'-3')** |
| --- | --- | --- |
| KLF1 | TGTCTGGGACCTGGTGCGGT | AAGGCTTCTCTCCGGTGTGCGT |
| KLF3 | AGCGGCATGCCAGTACCCGT | CCGGGCTGCACAATGACGGA |
| KLF4 | ACGGCAGCCACCCTGTGGTA | GTGTTGGGCCTGTGGCCGTT |
| KLF5 | CCAGAACTGAGCACTGCGCAC | GCGACTGCCTGTCTGGAGATGA |
| KLF6 | acgaccaagtttacctctgac | cagccccatagttgagaagat |
| KLF7 | AGTGGCGTTTTGCACGGAGCG | GACCTGGAGAAACACCTGTCGC |
| KLF9 | GGACGCGGGAAGAAGTGTCTT | CACGTGGCGGTCGCAAGTTT |
| KLF10 | TCTCCAGCAAGCTTCGGAGG | CGGTCTGAAGGTCAGGCGTC |
| KLF11 | ccaagtaatcaattcccaagg | aggctgacaggaaaccaagtt |
| KLF12 | CCACCTGGCACTGCACCGTA | TCAGGCACCGTGGGAATGGC |
| KLF15 | GCGTCAAGGTCGCCCAGCTT | GGCACAGGCGCAATTCGCAC |
| KLF16 | GGATTACTTTGCCGCCGACGT | GCCTCTCAGATCGGCCAGGAT |
| Bcl2 | CACGGTGGTGGAGGAACTCTT | GGTGACATCTCCCTGTTGACG |
| Bax | TGGAAGAAGATGGGCTGAGGC | CATTCCCACCCCTCCCAATAAT |
| Fas | ATGGCTGTCCTGCCTCTGGT | ACGCTCCTCTTCAACTCCAAA |
| IL-10 | gctatgttgcctgctcttactg | tctggctgactgggaagtg |
| TNF | gtgcctcagcctcttctcatt | ctctgcttggtggtttgctac |
| TNFSF12 | ggcctcgaagaagtgtttctaa | ggagctgttgattttggtctct |
| PYCARD | ccaaccaaaacaagatgagga | tctgtcaccaagtagggctgt |
| GAPDH | GGCTCTCTGCTCCTCCCTGTT | CTGTGCCGTTGAACTTGCCG |
